# Successful Management of Severe COVID-19 in a Kidney Transplant Recipient Safe Co-Administered Tacrolimus and Ensitrelvir: A Case Report

**DOI:** 10.3390/reports9020159

**Published:** 2026-05-19

**Authors:** Noriko Miyagawa, Satoshi Yamanouchi, Hideaki Fujimoto, Eichi Uchikanezaki, Yoshinobu Kameyama, Yugo Ashino, Toshio Hattori

**Affiliations:** 1 Department of Emergency, Sendai City Hospital, Sendai 982-8502, Japan; miyagawa-nor@hospital.city.sendai.jp (N.M.); manta3104@gmail.com (S.Y.); 2 Department of Pharmacy, Sendai City Hospital, Sendai 982-8502, Japan; fujimoto-hid@hospital.city.sendai.jp (H.F.); uchikane-h@hospital.city.sendai.jp (E.U.); 3 Department of Anesthesiology, Sendai City Hospital, Sendai 982-8502, Japan; y-kameyama@umin.ac.jp; 4 Department of Respiratory Medicine, Sendai City Hospital, Sendai 982-8502, Japan; 5 Roken Nursing Home, Akane, Kurashiki 712-8057, Japan

**Keywords:** COVID-19, kidney transplantation, tacrolimus, ensitrelvir, drug–drug interaction

## Abstract

**Background and Clinical Significance**: COVID-19 may worsen in patients receiving immunosuppressants. Furthermore, drug–drug interactions and concomitant use of anti-inflammatory drugs complicate treatment. We report the clinical course of severe COVID-19 pneumonia in a 74-year-old Japanese male kidney transplant recipient. **Case Presentation**: The patient had been taking tacrolimus (TAC) (2.5 mg/day), mycophenolate mofetil (1000 mg/day), and prednisone (5 mg/day) since his kidney transplant 7 years earlier. Twenty days before admission, he tested positive for SARS-CoV-2 antigen and was administered molnupiravir for 5 days. At admission, real-time PCR testing of a nasopharyngeal specimen revealed high viral loads, with Ct values of 22.2 and 27.9 for the *E* and *N2* genes, respectively. An oxygen flow rate of 15 L/min was required to maintain arterial oxygen saturation above 90%. TAC was continued, and antibiotics, steroids, anti-interleukin-6 receptor antibodies, intravenous immunoglobulin, and ensitrelvir (ESV) were administered. With invasive positive-pressure ventilation, positive end-expiratory pressure (PEEP), and prone positioning, the arterial oxygen tension/inspired oxygen tension (P/F) improved from 61.3 to 386 within 7 h. The patient was extubated 30 h after admission. The TAC dose was adjusted from 2.5 mg/day to 1 mg/day to achieve the target trough level. The patient was discharged on hospital day 8. PCR testing at discharge showed a decrease in viral load. **Conclusions**: This study provides insights into the treatment of COVID-19 in patients receiving immunosuppressants. Combination therapy of ESV and TAC was feasible in kidney transplant recipients with dose adjustment. The use of other anti-inflammatory drugs should also be considered.

## 1. Introduction and Clinical Significance

The novel coronavirus, Severe Acute Respiratory Syndrome Coronavirus 2 (SARS-CoV-2), has been spreading globally from one city in Hubei Province, China, to the whole world since December 2019 [1]. The COVID-19 pandemic ended as a Public Health Emergency of International Concern (PHEIC) in May 2023, as declared by the World Health Organization (WHO) [2]. However, in reality, the measures are insufficient, and the infection is spreading here and there [3]. Age is the strongest risk factor for severe COVID-19 outcomes. Patients with one or more underlying medical conditions are also at higher risk [4]. One reason for this is the use of corticosteroids or other immunosuppressive medications.

In transplant patients, TAC and Mycophenolate mofetil (MMF) are used to prevent post-transplant rejection. TAC acts by binding to the immunophilin FKBP-12 (FK506-binding protein) to form a new complex and reduce peptidyl-prolyl isomerase activity, thereby inhibiting both T lymphocyte signaling and IL-2 transcription [5]. MMF is known to suppress lymphocyte proliferation and the expression of T cell surface antigens, and to suppress rejection reactions in peripheral blood T cells [6]. Thus, solid organ transplant recipients are particularly vulnerable to severe acute respiratory syndrome coronavirus 2 (SARS-CoV-2) infection (COVID-19) due to their long-term immunosuppressive therapy [7]. At the same time, the use of calcineurin inhibitors (CNI) or mycophenolic acid derivatives may be a strategy to reduce viral replication. However, the report also notes that there is no clinical evidence on the potential benefits of these immunosuppressive drugs in transplanted patients with COVID-19 [8]. On the other hand, some argue that patients who have received solid organ transplants are inherently at risk of severe complications (associated complications include advanced age, hypertension, hyperlipidemia, diabetes, liver damage, and kidney damage, etc.) [8]. Antiviral therapy is generally recommended for COVID-19 patients at high risk of severe disease [9]. However, calcineurin inhibitors such as TAC are key drugs in post-transplant management, but are highly susceptible to pharmacokinetic interactions via the cytochrome P450 (CYP) 3A pathway. Consequently, treatment is restricted to oral or intravenous nucleoside analog administration in solid organ transplant recipients with COVID-19. If these are ineffective, the disease progresses to a severe stage. In such cases, it remains unclear whether immunosuppressive therapy should be continued, whether additional steroid administration is appropriate, and what antiviral medication should be selected [10]. Ensitrelvir (ESV), an oral SARS-CoV-2 3CL protease inhibitor, has attracted attention as a potential treatment for COVID-19. However, because it inhibits CYP3A, its effect on TAC trough levels is unknown due to pharmacokinetic interactions, and clinical experience in transplant patient populations is still limited [11]. Here, we report a case of severe COVID-19 in a kidney transplant patient who had acute respiratory distress syndrome after SARS-CoV-2 infection and was placed on invasive ventilation. The TAC dose was successfully adjusted under intensive monitoring, and treatment with ESV was successful. This is the first reported case globally in which a kidney transplant patient with severe COVID-19 was successfully treated through the simultaneous administration of ESV and TAC, with careful monitoring of TAC trough level variations.

## 2. Case Presentation

A 74-year-old Japanese man was admitted with fever and acute hypoxemic respiratory failure. His medical history included living-donor kidney transplantation at 67 years of age for nephrosclerosis, percutaneous coronary intervention, and prior endoscopic surgery for early-stage gastric cancer at a hospital specializing in Kidney Transplantation. He was a non-smoker and consumed alcohol occasionally.

### 2.1. Medications

After transplant treatment, his maintenance immunosuppression consisted of TAC (2.5 mg/day), prednisolone (PSL 5 mg/day), and MMF (1000 mg/day). The blood trough level of TAC was stable at 4.34–6.61 ng/mL over the past year. Concomitant medications included aspirin, nifedipine, olmesartan, and atorvastatin for cardiovascular disease.

### 2.2. Present Illness & Physical Examination

Seven years after transplantation, the patient developed cough and rhinorrhea and tested positive for COV2 by antigen testing at a nearby hospital. He initially received molnupiravir for five days; however, his condition continued to deteriorate even after 14 days. He visited again and tested positive again of COV2, with pneumonia found on his X-ray. His fever (39.0 °C) and cough have not improved at all, even though he has taken the prescribed antibiotics (Lascufloxacin 75 mg). Therefore, he returned to the same hospital four days later for a third time, and his COV2 antigen test came back positive. His condition deteriorated, and he was emergently transferred to our hospital with high-grade fever (38.5 °C) and respiratory failure, requiring high-flow oxygen supplementation. His oxygen saturation (SpO_2_) was 90% on a non-rebreather mask (NRM) at 15 (L/min). On admission, he appeared acutely ill. Lung auscultation revealed coarse crackles bilaterally, tachycardia (110 beats/min), blood pressure of 160/65 mmHg, and respiratory rate of 20 breaths/min.

### 2.3. Laboratory and Imaging Findings

**Figure 1:**
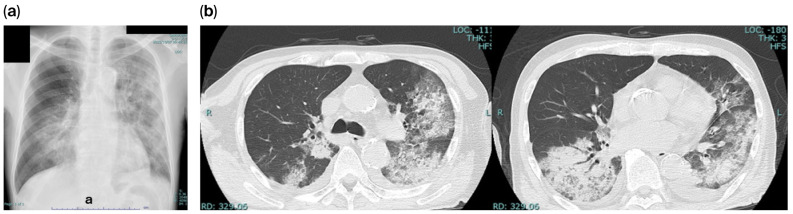
Chest X-ray and Computed tomography (CT) at the time of admission. (**a**) The X-ray images showed ground-glass opacities in both lungs, with infiltrates within them. (**b**) Chest CT images showed ground-glass opacity (GGO) with a crazy-paving pattern as the main feature in the upper lobes, accompanied by partial infiltrative shadows within them. In the lower lobes, ground-glass opacity was partially observed, but consolidation was predominant. Significant bilateral pleural effusions were seen.

The patient’s laboratory testing (Table 1) showed elevated inflammatory markers, including C-reactive protein (CRP 19.85 mg/dL) and ferritin (550 U/L), with neutrophil-predominant leukocytosis and lymphopenia. Renal function was mildly impaired (creatinine, Cre 1.35 mg/dL). Chest radiography and computed tomography (CT) scan demonstrated bilateral pulmonary infiltrates consistent with severe COVID-19 pneumonia (Figure 1). Furthermore, the CT scan showed bilateral pleural effusions, ground-glass opacities (GGOs), and a crazy-paving pattern around the consolidation in his left lung. In the right lung, significant consolidation was observed in segments 8 and 9. Polymerase chain reaction (PCR) testing and antigen testing of the patient’s nasopharyngeal swab confirmed CoV2 infection, while extensive testing for other viral, bacterial, and fungal pathogens was negative (Table 2). The PCR was performed using an assay targeting both the E and N genes of SARS-CoV-2. Cycle threshold (Ct) values for the E and N gene assay were 22.2 and 27.9, respectively. We decided to treat the patients as if they had critical conditions.

### 2.4. Clinical Course and Management (Figure 2)

The patient was immediately brought to the ICU and required endotracheal intubation and mechanical ventilation. In addition, treatment included tocilizumab (400 mg), intravenous immunoglobulin 5 g (for 3 days), dexamethasone 6.6 mg (for the first 7 days) 1.65 mg (the following 2 days), anticoagulation with heparin sodium at 10,000 uites (for 7 days), broad-spectrum antimicrobials levofloxacin (500 mg at one day one, followed by 250 mg daily for up to 3 additional days), Linezolid 600 mg intravenously twice daily (for 5 days) and Meropenem Hydrate 0.5 g intravenously twice daily(for 9 days). After confirming the presence or absence of pathogens suggestive of an infectious disease, antibiotics were promptly discontinued (Table 2). Given the insufficient response to prior nucleoside analog therapy, the oral protease inhibitor ESV was initiated. ESV was administered at 375 mg on the first day and continued at 125 mg for 8 days until discharge (Total 9 days). At the previous hospital, the TAC trough value was 4.89 ng/mL at a daily dose of 2.5 mg. At admission, the value was 6.70 ng/mL. Following the introduction of ESV, the TAC dose was changed to 2 mg. Still, the TAC trough value increased approximately threefold (19.7 ng/mL) on day 1, prompting immediate dose adjustment and frequent therapeutic drug monitoring. Therefore, the TAC dosage was adjusted to 0.5 mg in the afternoon of day 2, for a total of 1.5 mg, and from day 3, 0.5 mg was administered twice daily, for a total of 1 mg. The optimal TAC Trough Level; Target Range (5.0–6.9 ng/mL [12]) was reached by the trough values measured on days 4 and 6, which ranged from 8.8 to 6.1 (Figure 3). With careful management, TAC levels were stabilized without evidence of acute rejection or drug-related toxicity.

**Figure 2:**
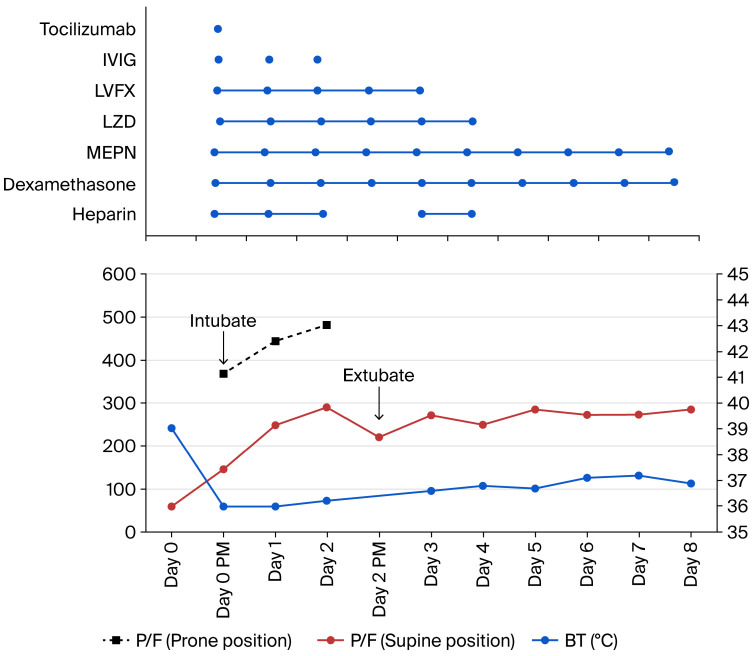
Clinical course of patients. Fluctuations in the patient’s body temperature and the P/F ratio during hospitalization. The duration of administered medications are detailed in the top section of the illustration. The data up to Day 2 represent the period of an artificial ventilator in the ICU. The ICU stay lasted until Day 6. The black squares and the dotted line show the P/F ratio and its time-course changes during prone positioning. IVIG: Intravenous Immunoglobulin; LVFX: Levofloxacin; LZD: Linezolid; MEPM: Meropenem; P/F: arterial oxygen tension/inspired oxygen tension.

**Figure 3:**
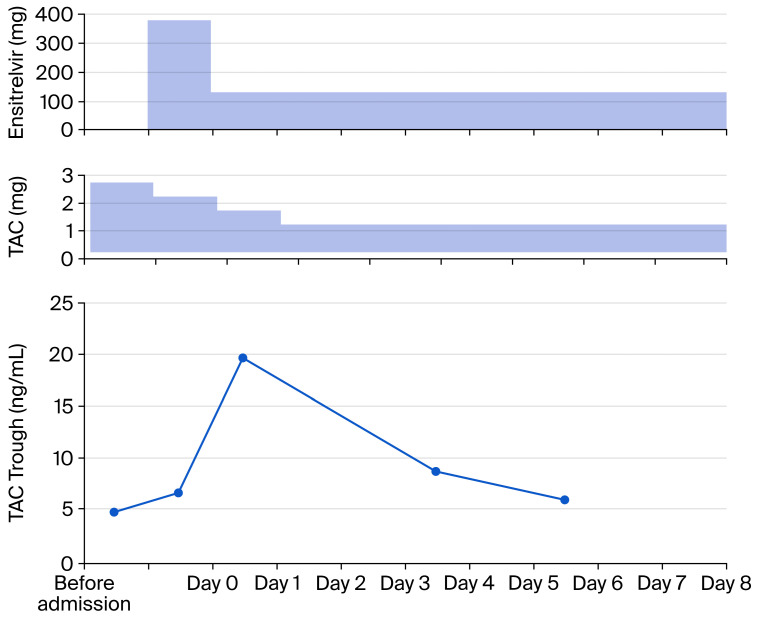
Interaction between Ensitrelvir (ESV) & Tacrolimus (TAC). The upper chart shows the daily changes in ESV dosage. The middle chart shows the daily changes in TAC dosage. The lower section indicates the changes in TAC trough values.

**Figure 4:**
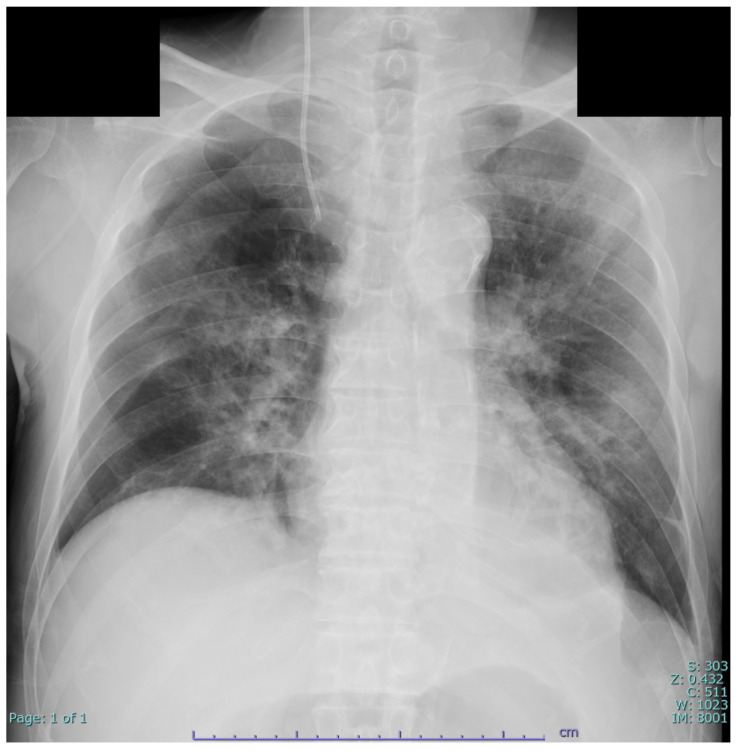
Chest radiograph obtained on day 6. While ground-glass opacities (GGOs) have resolved, reticular shadows persist in bilateral lungs.

The ventilator (Evita^®^ Infinity^®^ V500 Dräger, Lübeck, Germany) was set to Pressure Control-Assist/Control (PC/AC) mode with positive airway pressure (initial positive inspiratory pressure) of 15 cm H_2_O and an expiratory positive airway pressure of 10 cm H_2_O) and a tidal volume of 500 mL as Volume Guarantee under the condition of the fraction of inspiratory oxygen (FIO_2_) of 0.8. After 2 h, arterial blood gas analysis reported an arterial O_2_ tension (PaO_2_) of 117.0 Torr and an arterial CO_2_ tension (PaCO_2_) of 34.63 Torr. The change in the prone position was accompanied by a significant improvement in PaO_2_/FIO_2_ 231. Respiratory status differed depending on position. After being placed on a ventilator, the patient’s respiratory status gradually improved, allowing successful extubation on day 2. After that, he maintained a P/F of nearly 300 on 3 L of oxygen via nasal cannula. Serial SARS-CoV-2PCR testing showed increasing cycle thresholds for the E and N gene assays, consistent with declining viral load. However, neither Ct value showed virus levels below the detection limit (Table 3). Follow-up chest radiography showed marked improvement in pulmonary infiltrates (Figure 4). On day 9, the patient was transferred and discharged to the hospital, where he underwent a kidney transplant.

## 3. Discussion

In this report, we present a case in which simultaneous TAC and ESV therapy was initiated in a kidney transplant patient, resulting in good outcomes. To date, two cases have been reported in which ESV was used to treat SARS-CoV-2 infection in transplant or rheumatoid arthritis patients receiving TAC. However, in both cases, elevated TAC blood levels were observed, necessitating discontinuation of TAC treatment [11,12]. Based on these reports, we inferred that administering ESV increases TAC blood levels threefold and were able to treat critical COVID-19 cases by reducing the TAC dose and maintaining TAC blood levels within the therapeutic range. To our knowledge, this represents the first reported case of successful remission in a kidney transplant patient with severe COVID-19 treated with a combination of TAC and ESV.

ESV is a substrate of CYP3A and P-glycoprotein (P-gp) and an inhibitor of CYP3A, P-gp, breast cancer resistance protein (BCRP), and organic anion transporter 3 (OAT-3) [13]. However, the difference in the area under the blood concentration time curve (AUC) increase in midazolam (14.3-fold and 6.7-fold, respectively) between nirmatrelvir/ritonavir and ESV both 3CL protease inhibitors, when coadministered with midazolam, a pure CYP3A substrate, is likely due to the difference in the intestinal CYP3A inhibitory potency of NMV/RTV and ESV, with ESV’s inhibitory potency being considered weaker than NMV/RTV [11]. The impact on blood TAC concentrations varies depending on the concomitant medication [13]. In the case of ESV, it can be considered a viable option even when combined with TAC, provided the TAC dosage is reduced to 1/4 to 1/3 of the original dose [11,12]. The predicted increase in TAC AUC with concomitant use of ESV can be calculated based on pharmacokinetic considerations [14]. However, in actual clinical practice, TAC levels are affected by various factors, including the concomitant use of dexamethasone (a CYP3A4 inducer) [15] and proton pump inhibitors [16], as well as changes in liver function, hematocrit (Hct) levels, dietary intake, the presence of inflammatory conditions, and CYP3A5 genetic polymorphism [17,18,19]. Initially, we reduced the TAC dosage by 20%, anticipating a drug–drug interaction with ESV. However, the TAC trough level subsequently rose to 3 times baseline (pre-ESV). Previous reports indicate that the TAC dosage should be reduced to 1/3 or 1/4 of the original dose when combined with ESV [11,12], as in this case.

This was consistent with the present case. Therefore, when the TAC dose was reduced from 2.5 mg to 1 mg, the trough level approached the ideal target value. In this case, continuous monitoring of the TACs trough level enabled safe co-administration and prevented nephrotoxicity and rejection. In real-world clinical practice, caution is needed as the influence of other medications increases uncertainty. Ideally it is recommended to the need for trough level measurements every 24–48 h and dynamic dose adjustments, in order to prevent complications such as nephrotoxicity, neurotoxicity, or graft rejection.

The patient initially presented with cold-like symptoms, but developed a fever on day 17 of onset. On day 20, the fever and respiratory status rapidly deteriorated, and abnormalities were detected on lung imaging. Due to the worsening respiratory condition, the patient was intubated and placed on a ventilator. Subsequently, the patient was placed in the prone position, and PEEP (positive end-expiratory pressure) was applied, leading to an immediate improvement in the P/F ratio. Compared with other reports, the time to onset of respiratory failure and the rapid improvement observed in this case differed from the clinical course of Acute Respiratory Distress Syndrome (ARDS), which is typically driven by decreased lung compliance due to increased vascular permeability [20,21]. Regarding the length of ICU stay, the mean duration for patients with non-COVID-19 ARDS was 22.7 days, showing no significant difference from that of patients with COVID-19-associated ARDS (21.2 days) [22]. Based on this finding, this case is considered to fall under the ‘Rapidly Improving ARDS’ phenotype of COVID-19-associated ARDS [23]. Rapidly improving COVID-19-associated ARDS is rare, and its mechanism remains unknown, as it does not necessarily correlate with a favorable prognosis [24].

The relationship between transplant patients and COVID-19 remains largely unresolved, with no conclusions being drawn at present. Many aspects are still unclear. An expert panel from the American Association for the Study of Liver Diseases (AASLD) stated that while post-transplant immunosuppression has not been a risk factor for mortality as it was in SARS (2002–2003) and MERS (2012–present), it is premature to comment on its relationship with COVID-19 [25].

Although the underlying mechanism behind the rapid recovery despite the critical condition remains unclear, this case report suggests the following hypothesis.

First, while the time to worsening of COVID-19 clinical symptoms varies, changes in chest CT findings peaked 10 days after symptom onset [26]. Possible reasons why it took time for the symptoms to become severe in this case include in vitro studies indicating that calcineurin inhibitors and mycophenolic acid derivatives suppress viral replication [8]. Furthermore, it was suggested that, in immunosuppressed patients, this could be explained by the possibility that a weakened immune response plays a protective role [27]. On the other hand, immunosuppressed patients are considered at higher risk of severe COVID-19 [28]. Immunosuppression has been suggested to be involved in delayed symptom onset and increased severity of the disease.

Second, the pathophysiology of SARS-CoV-2-caused ARDS is similar to that of other causes of ARDS. A reduction in absolute CD4+ T cell counts and elevated IL-6 levels significantly correlated with pulmonary lesion volume in severe cases [29]. In this case, the level was also elevated to CRP 19.85 MG/dL, which was a serious level [30]. Nevertheless, rapid remission is thought to result from the following mechanism. SARS-CoV-2 targets various cell types in the proximal airways and type II alveolar epithelial cells in the gas-exchange region of the distal lung, which may lead to a reduction in pulmonary surfactant [31]. Surfactant deficiency causes alveolar cell injury [32]. Prone positioning (prone ventilation) not only mobilizes the dorsal lung fields, expelling airway secretions and improving gas exchange, but also forces air into collapsed dorsal alveoli, stretching them. This is thought to promote surfactant secretion from type II pneumocytes [33] and reduce alveolar collapse. If this mechanism drives lung damage, COVID-19 patients under immunosuppression might recover rapidly without progressing to pulmonary fibrosis, distinguishing them from those with cytokine-storm-induced ARDS [34]. However, because persistent SARS-CoV-2 viremia causes cellular dysfunction, prompt elimination of the virus is essential to reverse this collapse. In immunocompromised patients, rapid and intensive viral suppression is required, even if initial symptoms are mild. In this case, the immunosuppressive agents likely delayed the onset of apparent pulmonary dysfunction. Furthermore, the low Ct values at admission suggest persistent high viremia, which ultimately led to the progression of severe disease.

Predicting the severity of COVID-19 is crucial for saving patient lives.

The biological response to COVID-19 involves numerous immune cells, including dendritic cells, T cells, B cells, and plasma cells, that contribute to the immune system’s ability to control SARS-CoV-2 infection. TAC is a potent calcineurin inhibitor that blocks the dephosphorylation of the nuclear factor of activated T-cells (NFAT). This reduces the expression of cytokines such as interleukin-2 (IL-2) in the nucleus, thereby suppressing T-cell proliferation and differentiation [35]. Therefore, in COVID-19 patients who have received TAC, the immune response differs from that of normal patients, suggesting that predicting disease severity becomes more difficult. Recently, galectin-9 (Gal-9) was found to be released from neutrophils in COVID-19 [36], and Gal-9/neutrophil axis was proposed to play a pivotal role in COVID-19 [37]. Gal-9 can be considered a new biomarker for evaluating rheumatoid arthritis activity and therapeutic effects, including TAC [38], as it is known to reflect the severity of COVID-19 [39]. The levels could also reflect the severity of COVID-19 under TAC treatment; however, this remains a hypothesis at present and requires further investigation.

The patient received anti-IL-6 therapy, steroids, immunoglobulin, and antibiotics; however, the efficacy of steroids has not been established. In this case, the administration of steroids was considered, as it may address the deficiency of alveolar type II cells [40]. While tocilizumab has the potential to suppress cytokine storms in severe COVID-19 pneumonia, it has been reported that the drug does not significantly improve mortality or clinical outcomes in critically ill patients with lung injury requiring mechanical ventilation [41]. The use of antibiotics in COVID-19 pneumonia is also uncertain. Broad-spectrum coverage, including MRSA, is selected by healthcare providers as both directed and empirical therapy when faced with a severely ill inpatient and diagnostic uncertainty regarding potential bacterial co-infection [42]. In immunosuppressed patients in whom bacterial infection cannot be ruled out, it was thought that broad-spectrum antibiotics could not be stopped until the causative bacteria were confirmed.

This paper reports a case of a kidney transplant recipient with COVID-19 who was treated with a combination of immunosuppressants and anti-SARS-CoV-2 drugs, resulting in a favorable outcome. The mechanism of ARDS may differ during immunosuppressant use; therefore, accumulating more cases is essential to establish optimal treatment strategies, including antibiotic and anti-inflammatory drug use.

Future prospective studies should be conducted to validate these observations.

## 4. Conclusions

ESV may represent a valuable therapeutic option for severe or refractory COVID-19 in kidney transplant recipients. However, significant drug–drug interactions with TAC can occur. Close therapeutic drug monitoring and prompt dose adjustment are essential to ensure safety. Further accumulation of case data is needed to establish standardized management strategies in this high-risk population.

## Figures and Tables

**Table 1:** Laboratory data on admission.

	Reference Range	Result
Complete Blood Cell Count and Differential		
White cell unit (/µL)	3700–8500	8900
Neutrophils (%)	44.0–68.0	**87.5**
Lymphocytes (%)	27.0–44.0	**5**
Monocytes (%)	3.0–12.0	4.5
Eosinophils (%)	0.0–10.0	0.5
Basophils (%)	0.0–3.0	0.0
Hematocrit (%)	42.0–53	30.1
Hemoglobin (g/dL)	13.5–17.5	**9.8**
Platelet count × 10^3^ (/µL)	150–355	193
Red cell count × 10^6^ (/µL)	3.90–5.30	3.50
Biochemical Test		
Urea nitrogen (mg/dL)	8–20	**24**
Creatinine (mg/dL)	0.65–1.07	**1.35**
Alanine aminotransferase (U/L)	3–40	19
Aspartate aminotransferase (U/L)	8–35	23
Lactate dehydrogenase (U/L)	124–222	**258**
Ferritin (ng/mL)	14–304	**550**
C-reactive protein (mg/dL)	0.00–0.30	**19.85**
Total protein (g/dL)	6.6–8.4	**5.2**
Albumin (g/dL)	3.8–5.2	**2.7**
Urine Routin Test		
Color	Yellow	Yellow
Clarity	Clear	Clear
Specific gravity	1.009–1.025	1.020
pH	4.8–7.5	6.5
Protein	-	**1+**
Sugar	-	**4+**
White cells	-	-
Red cell	-	-
Immunoserological Test		
Endotoxin	0.0–5.0	<3.5
(1→3)-β-D-glucan	0.0–11.0	<6.5
Procalcitonin	0.00–0.05	**0.43**
Coagulation Test		
prothrombin time (s)	10.0–13.5	13.1
Prothrombin activity (%)	80.0–120.0	107.3
PT-International Normalized Ratio	0.90–1.10	0.97
Activated Partial Thrombop lasting Time (s)	24.0–39.0	**46.5**
D-dimer (µg/mL)	0.00–1.00	**3.27**
Fibrinogen (mg/dL)	200–400	**894**

Bold indicates the data is not within normal range.

**Table 2:** Pathogenic Microorganisms Screening.

			Result
Respiratory pathogen panel test PCR ^1^ Nasal swab		SARS-CoV-2-PCR	+
		InfluenzaA-PCR	−
		InfluenzaB-PCR	−
		Adenovirus-PCR	−
		RS Virus-PCR	−
		Human Metapeuumo-PCR	−
		Myco pneumonia-PCR	−
Culture Test		sputum	−
		blood	−
		urine	−
Antigen test	blood	cytomegalovirus pp65 antigenemia ^2^	−
	Nasal swab	InfluenzaA ^3^	−
		InfluenzaB ^3^	−
		SARS-CoV-2 ^3^	+
	urine	Pneumococcal antigen ^4^	−

^1^ BioFire FilmArray Pneumonia plus Panel (bioMérieuxJapan Ltd., Tokyo, Japan). ^2^ The cytomegalovirus (CMV) pp65 test involved directly staining and counting cells for the PP65 antigen present in white blood cells (neutrophils) in the blood using the immunofluorescence method. ^3^ Rapid syndromic PCR testing was used by the SARS coronavirus and influenza antigen test is performed using the SARS-CoV-2 & Flu A/B Rapid Antigen Test (Roche Diagnostics K.K., Tokyo, Japan) via rapid immunochromatography. ^4^ Pneumococcal urinary antigen was measured using Pneumococcal Antigen Test Kit: BinaxNOW™ Streptococcus pneumoniae Antigen Card Abbott Diagnostics Medical Co., Ltd. Abbot, Tokyo, Japan.

**Table 3:** Pre- and post-treatment SARS-CoV-2 PCR Ct (Threshold Cycle).

Assay Primer/Probe	Reference Range	Pre	Post
Ct value Gene E	>40	22.2	28.2
Ct value Gene N2	>40	27.9	36.4

Real-time PCR was performed using the GeneXpert^®^ System (Beckman Coulter, Inc., Brea, CA, USA). The reaction mixture contained Xpert Xpress SARS-CoV-2 (Cepheid, Sunnyvale, CA, USA).

## Data Availability

Data are available from Y.A. at the Department of Respiratory Medicine, Sendai City Hospital. The data that support the findings of this study are available from the corresponding author, Y.A., upon reasonable request due to privacy concern.

## Abbreviations

The following abbreviations are used in this manuscript:

TAC	tacrolimus
ESV	Ensitrelvir
CYP	cytochrome P450
AUC	area under the blood concentration time curve
ARDS	Acute Respiratory Distress Syndrome
MERS	Middle East Respiratory Syndrome
